# Long glucocorticoid-induced leucine zipper regulates human thyroid cancer cell proliferation

**DOI:** 10.1038/s41419-018-0346-y

**Published:** 2018-02-21

**Authors:** Emira Ayroldi, Maria Grazia Petrillo, Maria Cristina Marchetti, Lorenza Cannarile, Simona Ronchetti, Erika Ricci, Luigi Cari, Nicola Avenia, Sonia Moretti, Efisio Puxeddu, Carlo Riccardi

**Affiliations:** 1 Department of Medicine, Section of Pharmacology, Medical School, University of Perugia, Perugia, Italy; 2 Department of Surgical and Biomedical Sciences, Medical School, University of Perugia, Perugia, Italy; 3 Department of Medicine, Section of Endocrinology, Medical School, University of Perugia, Perugia, Italy; 4 Present Address: Signal Transduction Laboratory, Department of Health and Human Services, National Institute of Environmental Health Sciences, National Institutes of Health, Research Triangle Park, NC USA

## Abstract

Long glucocorticoid-induced leucine zipper (L-GILZ) has recently been implicated in cancer cell proliferation. Here, we investigated its role in human thyroid cancer cells. L-GILZ protein was highly expressed in well-differentiated cancer cells from thyroid cancer patients and differentiated thyroid cancer cell lines, but poorly expressed in anaplastic tumors. A fusion protein containing L-GILZ, when overexpressed in an L-GILZ-deficient 8505C cell line derived from undifferentiated human thyroid cancer tissue, inhibited cellular proliferation in vitro. In addition, when this protein was injected into nude mice, in which cells from line 8505C had been transplanted, xenograft growth was reduced. Since the mitogen-activated protein kinase (MAPK) pathway is frequently hyperactivated in thyroid cancer cells as a result of the BRAF^V600E^ or Ras mutation, we sought to further investigate the role of L-GILZ in the MAPK pathway. To this end, we analyzed L-GILZ expression and function in cells treated with MAPK inhibitors. We used 8505C cells, which have the BRAF^V600E^ mutation, or the CAL-62 cell line, which harbors a Ras mutation. The cells were treated with the BRAF-specific drug vemurafenib (PLX4032) or the MEK1/2 inhibitor, U0126, respectively. Treatment with these agents inhibited MAPK activation, reduced cell proliferation, and upregulated L-GILZ expression. L-GILZ silencing reversed the antiproliferative activity of the MAPK inhibitors, consistent with an antiproliferative role. Treatment with MAPK inhibitors led to the phosphorylation of the cAMP/response element-binding protein (CREB), and active CREB bound to the *L-GILZ* promoter, contributing to its transcription. We suggest that the CREB signaling pathway, frequently deregulated in thyroid tumors, is involved in L-GILZ upregulation and that L-GILZ regulates thyroid cancer cell proliferation, which may have potential in cancer treatment.

## Introduction

Long glucocorticoid-induced leucine zipper (L-GILZ) is a transcriptional variant of the well-studied GILZ protein^1^, which is mainly induced by glucocorticoids (GCs) and mediates several anti-inflammatory and immunomodulatory GC-related functions^2,3^. In contrast, L-GILZ is involved in regulating cell differentiation and tumorigenesis by binding Ras^4–6^. We have recently demonstrated that L-GILZ exerts antiproliferative and anti-oncogenic activity by activating p53^5^, as interactions between L-GILZ, p53, and mouse double minute 2 (MDM2) led to the activation of p53 and inhibition of tumor cell growth^5,7^. To further investigate the role of L-GILZ in cancer cell development, we used several cell lines derived from human thyroid carcinomas at various grades of differentiation as a model system. The well-characterized genetic alterations of the cell lines are associated with phenotypes and biological characteristics relevant for this investigation^8^.

Thyroid cancer is an endocrine malignancy characterized by several genetic aberrations that produce different thyroid cancer isotypes. Its development and progression involve phenotype-specific gene mutations that affect cell differentiation, proliferation, and apoptosis^9^. The histopathological classification of thyroid tumors has several significant prognostic and therapeutic implications. Thyroid tumors are classified as follicular thyroid carcinoma (FTC), papillary thyroid carcinoma (PTC) (both characterized as differentiated thyroid carcinoma, DTC), and anaplastic thyroid carcinoma (ATC), which accounts for more than half of all thyroid cancer-related deaths^9,10^. Generally, a single specific genetic mutation leads to the initiation of a thyroid tumor with a corresponding histological type, although the same mutation can occasionally occur in diverse phenotypes. However, as the disease progresses, multiple genetic mutations can be associated with the same histopathological phenotype^11^.

The constitutive aberrant activation of mitogen-activated protein kinase (MAPK) signaling (also known as the RAS-RAF-MEK-ERK signaling pathway), which normally regulates physiological proliferative events, is frequently found in thyroid cancers. Mutations in proto-oncogenes (e.g., *BRAF*, *Ras*, and *Ret*) are often observed in DTC and ATC tumors; for example, Ras mutations have been identified in 60% of ATCs^9,12,13^. In contrast, p53 inactivation is found in up to 95% of ATCs and occurs late in the tumorigenic process^14^.

The BRAF^V600E^ point mutation in most PTCs induces the activation of BRAF kinase, which then hyperactivates the MAPK pathway, resulting in uncontrolled cellular proliferation and tumorigenesis^15,16^. This mutation is also linked to unfavorable clinical outcomes due to its association with de-differentiation characteristics, including the loss of the sodium iodide symporter (NIS), which mediates active iodide uptake, and the subsequent resistance to radioiodine ablation therapy^17^. Dysregulation of the thyroid-stimulating hormone (TSH)/TSH receptor (TSHR)/cAMP/PKA/cAMP response element-binding protein (CREB) signaling pathway, which plays an important role in thyroid development, also contributes to tumorigenesis and is found in most anaplastic thyroid cancers^18^. Recent advancements in thyroid cancer treatment includes targeted therapy with kinase inhibitor drugs, especially those that inhibit the abnormal oncogenic activation of MAPK pathway protein kinases^19^. Since most crucial signaling pathways are deregulated in thyroid tumors^11^ and L-GILZ affects cellular proliferation and tumorigenesis by interfering with Ras^4^ and p53 signaling^5,7^, we investigated the differential expression and function of L-GILZ in thyroid cancer cells treated with MAPK pathway inhibitors.

## Results

### L-GILZ is primarily expressed in highly differentiated thyroid cancer cells

**Fig. 1:**
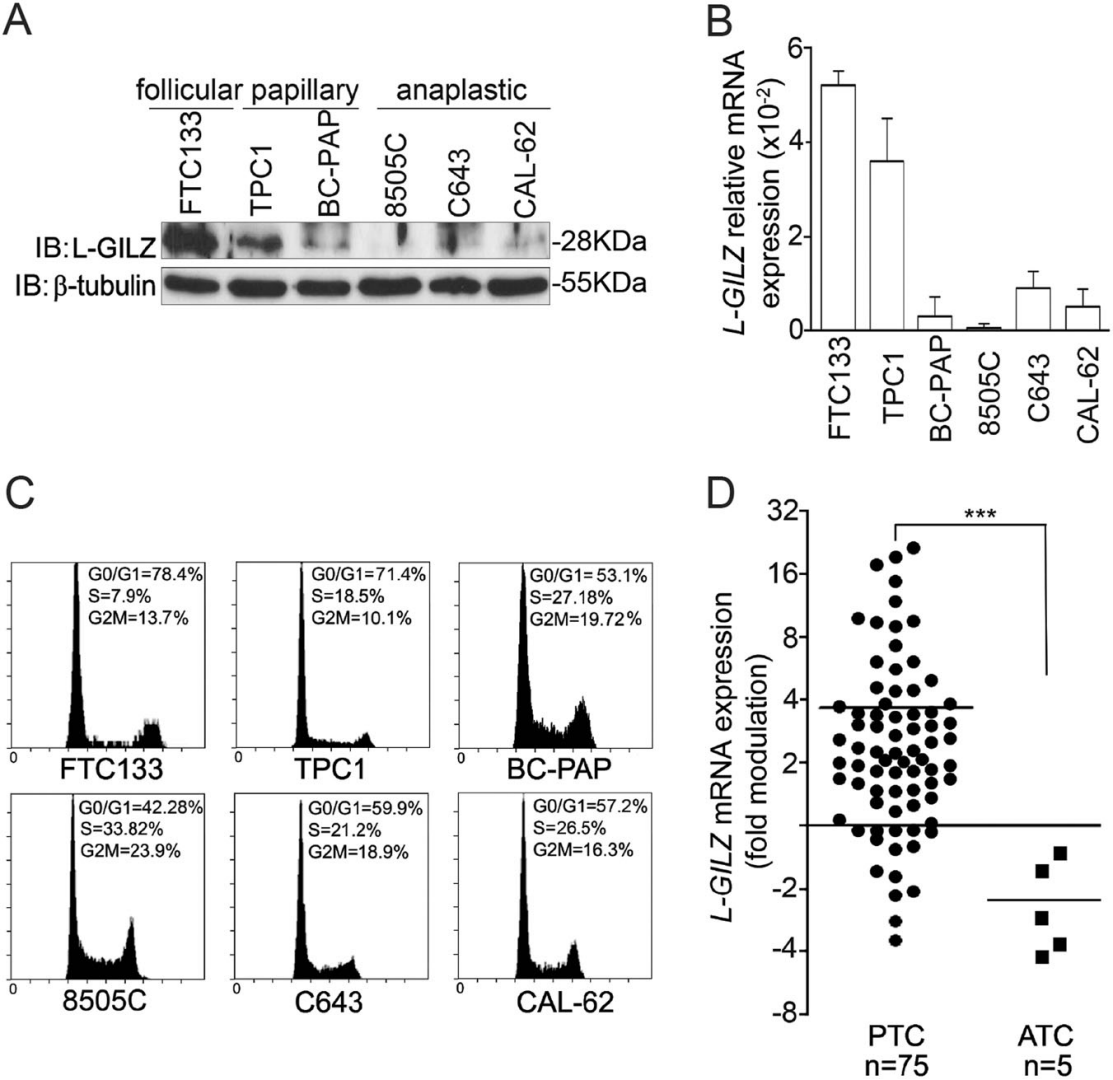
Endogenous L-GILZ is highly expressed in differentiated thyroid cancer cells. Representative data from **a** Western blots, **b** qRT-PCR, and **c** cell cycle PI assays of follicular, papillary, and anaplastic thyroid cancer cell lines. *L-GILZ* mRNA expression in the indicated thyroid cell lines is relative to the expression of *HPRT* mRNA. Panel **c** includes representative results (DNA content, *x*-axis; number of nuclei, *y*-axis). Data were obtained from triplicate experiments and expressed as the mean ± SD. **d** *L-GILZ* expression in surgical specimens from thyroid cancer patients is shown as the fold-modulation of relative mRNA levels in PTC (papillary) or ATC (anaplastic) tissues compared to those in a normal thyroid gland. The mean value (horizontal lines) of *L-GILZ* expression was significantly different in PTC and ATC tissues. ***p = 0.0006

To explore the role of L-GILZ in the proliferation of thyroid malignancies, we used thyroid tumor-derived cell lines in diverse stages of differentiation^8,20^ and first analyzed the level of endogenous L-GILZ expression. Western blotting showed that L-GILZ was primarily expressed in the more highly differentiated FTC-133 and TPC-1 cells, while its expression was undetectable in BC-PAP, 8505C, C643, and CAL-62 cells (Fig. 1a). Corroborating results were obtained by mRNA analysis using real-time polymerase chain reaction (PCR) (Fig. 1b). Although cellular proliferation and differentiation are associated with signaling pathways and genetic programs that can occasionally overlap, the majority of the time such pathways are separate. Moreover, it is generally accepted that a greater degree of tumor differentiation is accompanied by a lower proliferative index^21^. Indeed, PI staining revealed that FTC-133 and TPC-1 cells had a lower proliferative rate than anaplastic cancer cells (Fig. 1c).

We next evaluated L-GILZ expression in surgical specimens obtained from thyroid cancer patients. Real-time PCR analysis indicated that L-GILZ mRNA was more highly expressed in well-differentiated thyroid tumors than in anaplastic tumors (Fig. 1d). The small number of ATC samples analyzed in our study is justified by their comparative rarity^22^.

### Effects of sorafenib and PLX4032 on L-GILZ expression

**Fig. 2:**
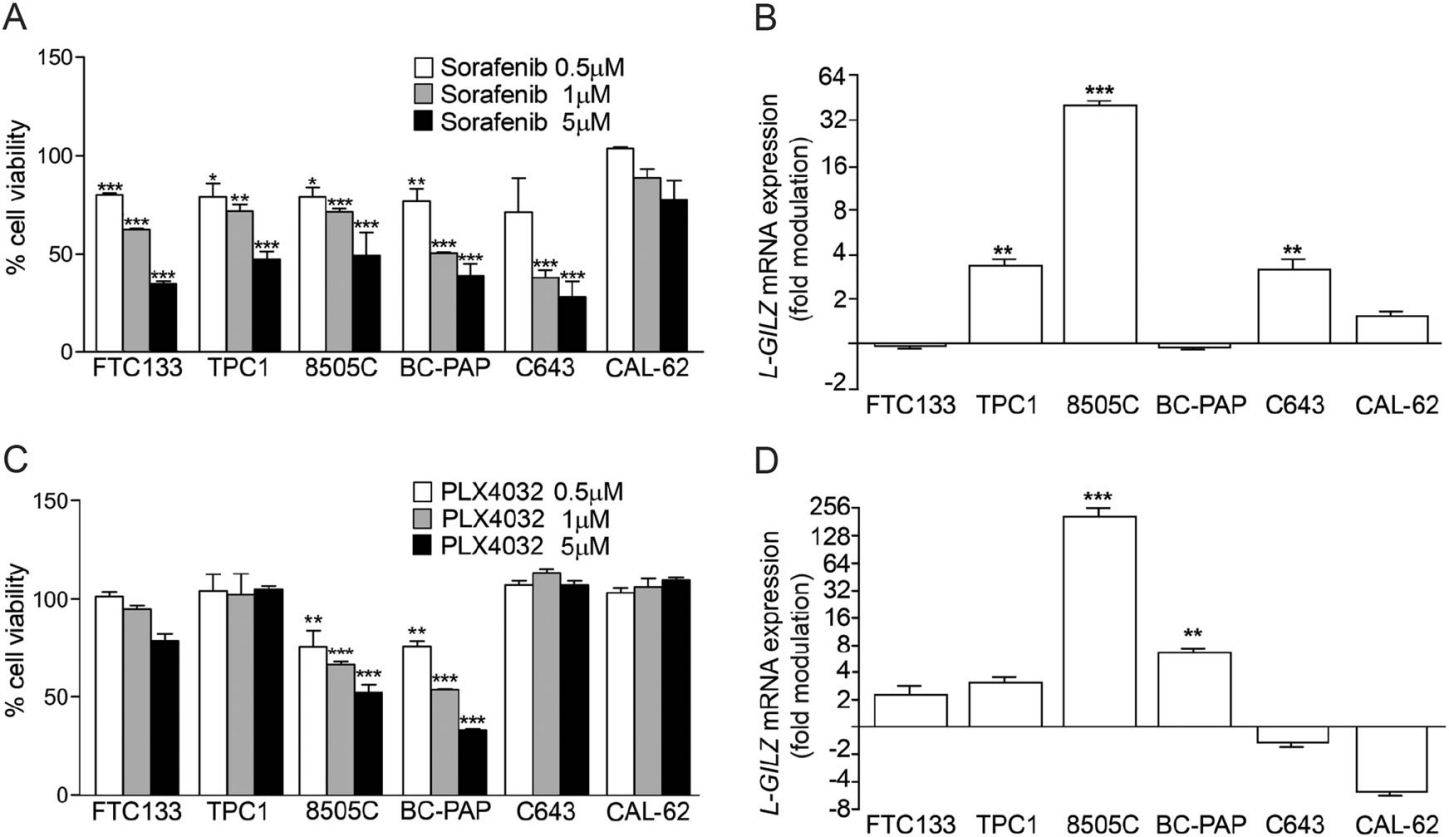
Effects of sorafenib and PLX4032 on thyroid cancer cell line proliferation and L-GILZ expression. The indicated cell lines were treated with different concentrations of sorafenib or PLX4032, and the viability of sorafenib-treated cells (**a**) and PLX4032-treated cells (**c**) was evaluated by trypan blue exclusion. Cell viability was expressed as the percentage of control (DMSO-treated cells) and ***, **, and * values describe the direct comparison of sorafenib/PLX4032-treated versus DMSO-treated cells. *L-GILZ* expression was evaluated by qRT-PCR in sorafenib-treated (**b**) and PLX4032-treated (**d**) cell lines and is presented as the fold-modulation of *L-GILZ* mRNA levels in drug-treated versus DMSO-treated cells. Data are representative of triplicate experiments

Kinase inhibitors have been recently approved for clinical use in thyroid carcinomas resistant to conventional therapies^23,24^. Sorafenib, a multi-target tyrosine kinase inhibitor targeting RAF, c-KIT, and RET kinases, has been shown to improve the progression-free survival of metastatic DTC patients resistant to radioactive iodine^25^. PLX4032 targets mutated BRAF^V600E^ and inhibits MAPK signaling^26^ and is effective in PTC patients harboring BRAF^V600E^ ^27,28^. Thus, we investigated the possible role of L-GILZ in the antiproliferative effects induced by these drugs. Sorafenib inhibited proliferation in all cell lines except CAL-62 (Fig. 2a), and upregulated L-GILZ in TPC-1, 8505C, and C643 cells, but not in FTC-133, BC-PAP, or CAL-62 cells (Fig. 2b). To elucidate the pathway(s) involved in L-GILZ upregulation, we next examined the effects of PLX4032. PLX4032 limited the proliferation of cell lines carrying BRAF^V600E^ ^29^ (Fig. 2c) and significantly induced the upregulation of L-GILZ in 8505C and BC-PAP cells, and moderately upregulated L-GILZ in FTC-133 and TPC-1 cells (Fig. 2d), suggesting a correlation between MAPK inhibition-mediated antiproliferative activity and the upregulation of L-GILZ. Notably, PLX4032 treatment of the undifferentiated cell lines (C643 and CAL-62) did not noticeably affect their proliferation or L-GILZ expression.

### L-GILZ contributes to the antiproliferative effects of MAPK inhibitors

**Fig. 3:**
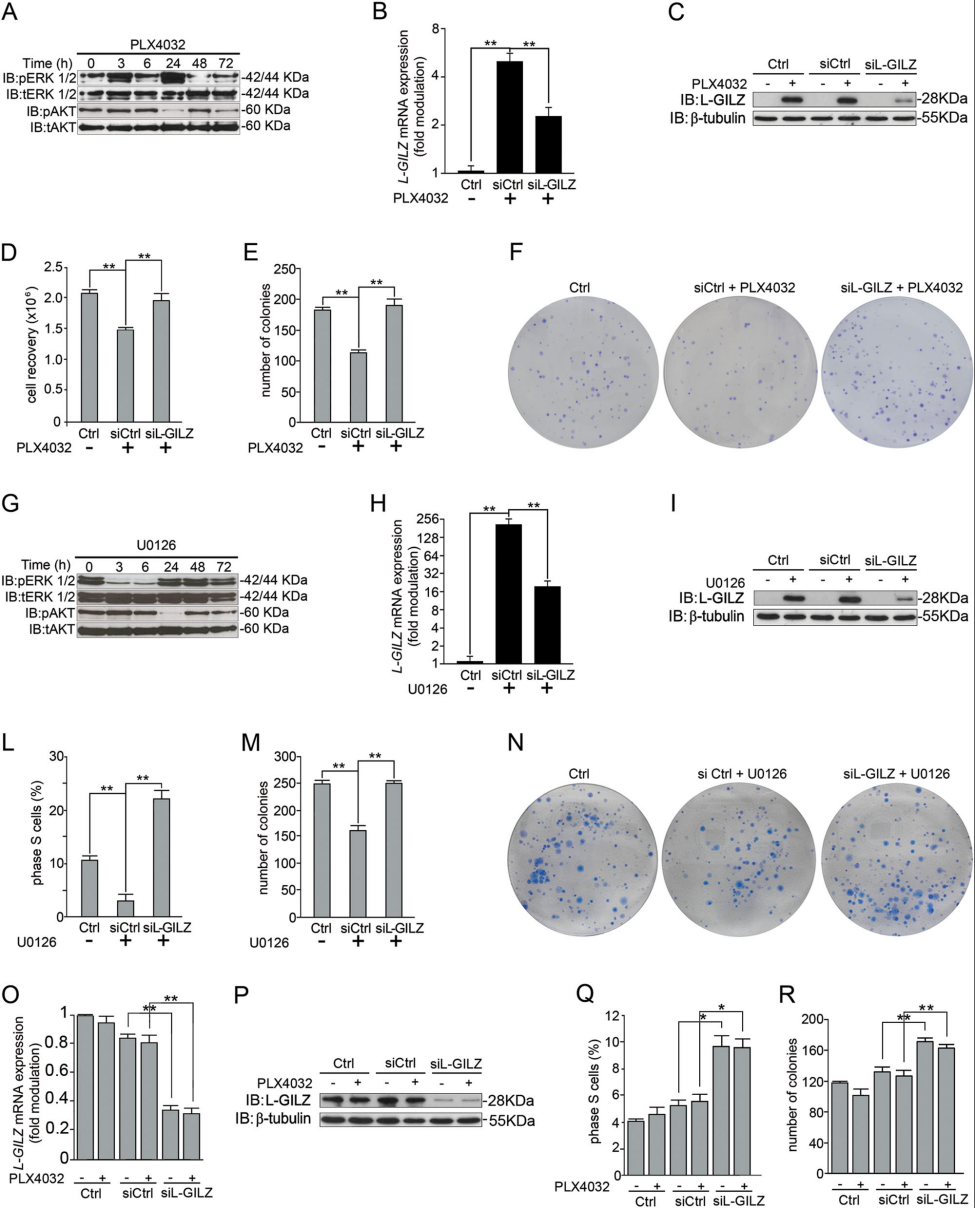
Silencing of L-GILZ decreases PLX4032-induced and U0126-induced antiproliferative effects. Lysates from undifferentiated 8505C and CAL-62 cell lines, treated with PLX4032 (**a**) or U0126 (**g**), respectively, for varying time periods or left untreated, were analyzed by immunoblotting with the indicated antibodies. The 8505C and CAL-62 cells were transfected with *L-GILZ* (siL-GILZ) or a scrambled control (siCtrl) siRNAs before adding PLX4032 (**b**, **c**) or U0126 (**h**, **i**), respectively. *L-GILZ* expression was measured by qRT-PCR (**b** and **h**) and Western blot (**c** and **i**). *L-GILZ* mRNA expression relative to *HPRT* mRNA expression is presented as the fold-modulation of the relative mRNA levels of either PLX4032-treated or U0126-treated cells compared to the relative mRNA levels of DMSO-treated control cells (Ctrl). After 48 h silencing, PLX4032-treated 8505C cells were counted by trypan blue exclusion (cell recovery, **d**) and U0126-treated CAL-62 cells were stained by PI for cell cycle analysis (**l**); histograms shown in **d** and **l** represent the average of either cell count or the percentage of cells in the S phase, respectively, of three independent experiments. PLX4032-treated 8505C (**e**) and U0126-treated CAL-62 (**m**) cells were plated in 10-cm dishes in triplicate for clonogenic assays. The number of colonies shown in **e** and **m** represents the mean ± SD of three independent experiments. Representative plates for each group are shown in **f** and **n**. **The direct comparison of siL-GILZ versus siCtrl cells and siCtrl versus Ctrl cells. The values of untreated-siL-GILZ cells (in qRT-PCR and clonogenic assay) were omitted because they were identical to those of non-silenced untreated control. TPC-1-differentiated cells were transfected with *L-GILZ* (siL-GILZ) or scrambled control (siCtrl) siRNAs before adding PLX4032 and *L-GILZ* expression was measured by qRT-PCR (**o**) and Western blot (**p**). *L-GILZ* mRNA expression relative to *HPRT* mRNA expression is presented as described in **b**. After 48 h silencing, the TPC-1 cells were stained with PI for cell cycle analysis (**q**) or plated in 10-cm dishes in triplicate for clonogenic assays (**r**). **The direct comparison of siL-GILZ versus control cells (Ctr) and PLX4032-siL-GILZ versus PLX-treated cells

To further investigate the role of L-GILZ in sorafenib-mediated and PLX4032-mediated inhibition of proliferation, we focused on the Raf/MEK/ERK pathway, which is inhibited by both drugs^28,30,31^. We excluded sorafenib for further investigation due to its lack of selectivity^25^ and focused on drugs that inhibit MAPK pathway. We selected PLX4032 for the treatment of 8505C cells and U0126, a MEK1/2 inhibitor, for the treatment of CAL-62 cells, which as seen in Fig. 2c, are PLX4032-unresponsive. Western blot data demonstrated that PLX4032 inhibited ERK and Akt phosphorylation in 8505C cells (Fig. 3a). In particular, after an initial 3-h hyperphosphorylation period, ERK phosphorylation was inhibited at 6, 48, and 72 h with a hyperphosphorylation rebound at 24 h. In contrast, Akt was inhibited at 24 and 72 h with a rebound at 48 h (Fig. 3a). To determine if L-GILZ plays a role in the antiproliferative effect of PLX4032, 8505C cells were treated with PLX4032, and *L-GILZ* was knocked down using specific small interfering RNA (siRNA). PLX4032 upregulated L-GILZ mRNA (Fig. 3b) and protein (Fig. 3c) and significantly reduced the number of viable cells (cell recovery) and colonies formed (Fig. 3d–f). L-GILZ silencing, confirmed by PCR and Western blot (Fig. 3b, c), blocked the effect of PLX4032 and restored the PLX4032-induced reduction of both total cell and colony numbers (Fig. 3d–f). To validate that the upregulation of L-GILZ is mediated via the inhibition of the MAPK pathway and plays a role in the antiproliferative activity of drugs inhibiting MAPK, we used the CAL-62 cell line, which does not respond to PLX4032, but since its carries a hyperactivating Ras mutation, still exhibits abnormal activation of the MAPK pathway. Thus, CAL-62 cells were treated with U0126, which inhibits MAPK signaling downstream of BRAF^32^. U0126 induced the early inhibition of ERK phosphorylation (3–6 h) and the later inhibition of Akt (24 h) (Fig. 3g). U0126 also upregulated L-GILZ mRNA and protein expression (Fig. 3h, i), reduced the percentage of cells in the S phase (Fig. 3l), and decreased the number of colonies formed (Fig. 3m, n). Moreover, L-GILZ silencing (Fig. 3h, i) restored cellular proliferation (Fig. 3l–n). Finally, we evaluated the effects of L-GILZ silencing on the differentiated cell line, TPC-1, which as shown in Fig. 1, constitutively expresses L-GILZ. The cells were treated with PLX4032 or left untreated, and L-GILZ silencing, established by PCR and Western blot (Fig. 3o, p), induced an increase in the number of cells in the S phase of the cell cycle (Fig. 3q) and an augmentation of the number of colonies (Fig. 3r) both in the untreated control and PLX4032-treated cells.

Overall, these data suggest that L-GILZ exerts antiproliferative activity on differentiated thyroid cancer cells and that, when upregulated by MAPK inhibitors in anaplastic thyroid cancer cell lines, contributes to their antiproliferative activity.

### MAPK inhibitors, PLX4032 and U0126, induce CREB phosphorylation

**Fig. 4:**
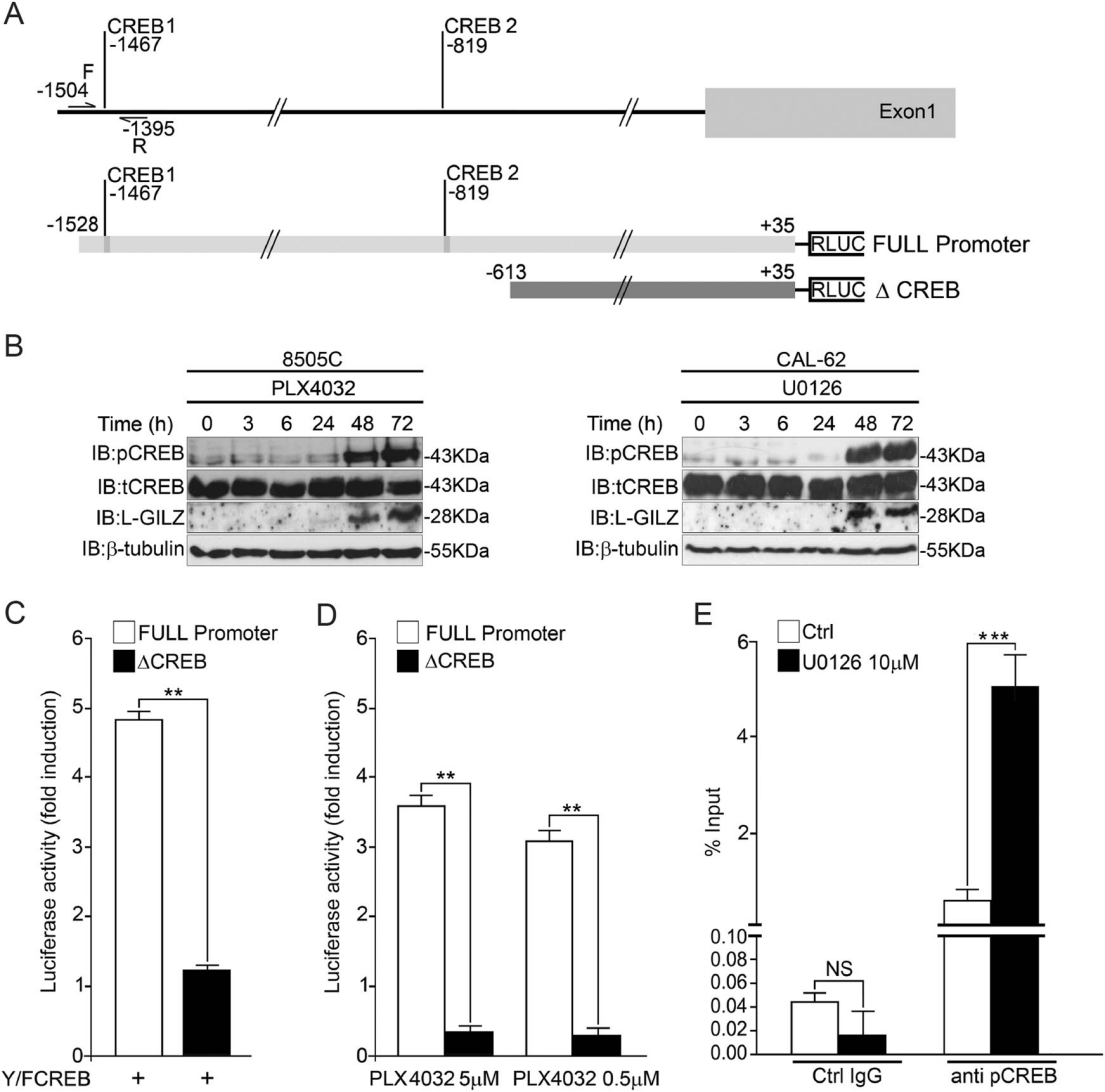
Phosphorylated CREB binds and transactivates the *L-GILZ* promoter. **a**, Upper lane: a schematic of the human *L-GILZ* promoter region. The two putative CREB-binding sites (CREB1 starting at −1467 and CREB2 starting at −819) were identified. Forward (F) and reverse (R) primers for CREB1 were used. Lower lanes: a schematic of the luciferase reporter gene constructs containing the *L-GILZ* promoter, including both CREB1 and CREB2 sites (FULL promoter +35/−1528) or the *L-GILZ* promoter lacking these sites (ΔCREB +35/−613). **b** The 8505C and CAL-62 cell lines were treated for the indicated time periods with PLX4032 and U0126, respectively, and the cell lysates were analyzed by Western blot using anti-p-CREB, anti-CREB, anti-L-GILZ, and anti-β-tubulin antibodies. **c** The HEK 293 cell line was transfected with either the FULL promoter or ΔCREB luciferase reporter construct together with activated CREB (Y/F CREB plasmid) or an empty vector. The cells were harvested 48 h post transfection, and transcription activation was assessed as the fold-induction of luciferase activity of Y/F CREB-transfected over empty vector**-**transfected cells. **d** The 8505C cells were transfected with either the FULL promoter or ΔCREB and treated with PLX4032. Activation of transcription was assessed as the fold-induction of luciferase activity in PLX4032-treated cells versus DMSO-treated cells. **Value describes the direct comparison between the FULL promoter-transfected and ΔCREB-transfected cells. **e** For ChIP analysis, equivalent amounts of chromatin from CAL-62 cells treated with U0126 or DMSO (control) were immunoprecipitated with anti-p-CREB or control IgG antibodies. The immunoprecipitated samples were analyzed by qRT-PCR using F and R primers for CREB1. Results from three independent ChIP experiments are expressed as the percentage of input chromatin. ***Value describes the direct comparison between treated and untreated cells

To investigate the signaling pathways responsible for L-GILZ upregulation following treatment with PLX4032 or U0126, we examined the genomic sequence upstream of the L-GILZ coding region. Among the putative binding sites for the transcription factors involved in L-GILZ upregulation, we focused our attention on the binding sites of CREB (CREB1 and CREB2; Fig. 4a), a transcription factor downstream of the TSH/TSHR/cAMP-dependent PKA pathway that regulates TSHR and NIS^33,34^.

We first evaluated CREB phosphorylation in 8505C and CAL-62 cell lines treated with PLX4032 and U0126, respectively, in a time-course experiment. Both drugs induced CREB phosphorylation and L-GILZ expression at 48 and 72 h (Fig. 4b). *L-GILZ* promoter reporter plasmids containing either the two putative CREB-binding regions (FULL promoter) or lacking such regions (ΔCREB, control) were constructed (Fig. 4a) and co-transfected into human embryonic kidney 293 (HEK 293) cells with the constitutionally activated CREB plasmid (Y/F CREB)^35^. As shown in Fig. 4c, the FULL promoter, but not ΔCREB, exhibited enhanced transcriptional activity when co-transfected with Y/F CREB compared to the group transfected with an empty vector. Since CREB transcriptional activity is associated with its phosphorylation, we evaluated the activity of the FULL promoter and ΔCREB in 8505C cells treated for 48 h with PLX4032, which induces CREB phosphorylation (Fig.  4b). The FULL promoter, but not ΔCREB, exhibited transcriptional activity when treated with both doses of PLX4032 (Fig. 4d).

Finally, to assess the direct action of phosphorylated CREB on L-GILZ transcription, we performed chromatin immunoprecipitation (ChIP) assays which revealed that in CAL-62 cells treated with U0126 for 48 h, p-CREB was strongly recruited to CREB1 (Fig. 4a). The pull-down was specific for p-CREB, as no significant enrichment was detected using the control antibody (Fig. 4e). Taken together, these data suggest that inhibition of the MAPK pathway induces the phosphorylation of CREB, which subsequently binds and activates *L-GILZ* promoter transcriptional activity and induces L-GILZ expression.

### p38, but not PKA, is involved in CREB phosphorylation and L-GILZ upregulation

**Fig. 5:**
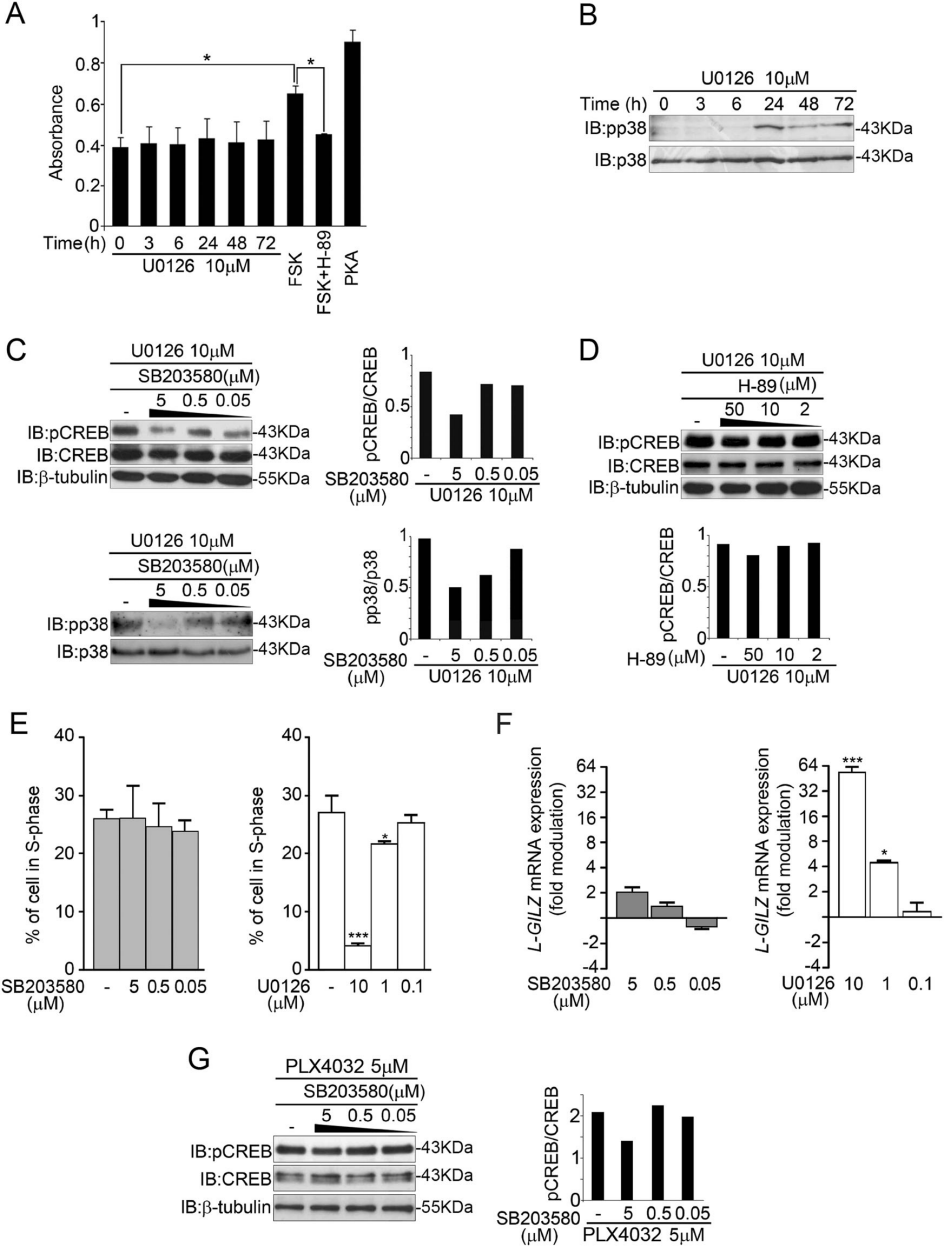
p38 involvement in CREB phosphorylation and L-GILZ upregulation. **a** CAL-62 cells were treated for the indicated time periods with U0126, and the cell lysates were measured for PKA activity via ELISA. The cells were treated with only forskolin (FSK) or FSK and H-89. Cells treated with active PKA served as a positive control. **b** Lysates from CAL-62 cells treated for the indicated time periods with U0126 were analyzed by Western blot with anti-p-p38 or anti-p38 antibodies. **c**, **d** Lysates from CAL-62 cells, pretreated with U0126 and subsequently treated with either SB203580 (**c**) or H-89 (**d**) at the indicated doses, were analyzed by immunoblotting using p-CREB, CREB, β-tubulin, p38, and p-p38 antibodies. Histograms present the ratios of p-CREB/CREB and p-p38/p38 determined by a densitometry analysis of each immunoblot. **e** CAL-62 cells were treated with different concentrations of SB203580 or U0126, and the resulting cell cycle staging was evaluated by PI staining. **f** *L-GILZ* expression was assessed by qRT-PCR and is presented by the fold-modulation of *L-GILZ* mRNA levels in drug-treated versus DMSO-treated cells. Data were obtained from triplicate experiments. ***, * The direct comparison between treated and untreated cells. **g** Lysates from 8505C cells, pretreated with PLX4032 and subsequently treated with SB203580 at the indicated doses were analyzed by Western blot using p-CREB and CREB antibodies. Histograms present the ratios of p-CREB/CREB by a densitometry analysis of each immunoblot

cAMP/PKA is the major pathway involved in CREB phosphorylation, and crosstalk between the Ras/MAPK/ERK and the cAMP/PKA pathways has long been recognized^36,37^. Therefore, we explored whether PKA is involved in CREB phosphorylation following MAPK inhibition. Unexpectedly, as shown in Fig. 5a, the enzymatic activity of PKA, evaluated in a time-course experiment, was not affected by U0126 treatment. We then focused on the p38-dependent pathway, which is also involved in CREB activation^38,39^. Inhibition of MAPK in CAL-62 cells by U0126 resulted in the phosphorylation of p38, which was first detected 24 h after treatment and was sustained for up to 72 h (Fig. 5b). If p38, but not PKA, is responsible for CREB activation, the pharmacological inhibition of p38 phosphorylation should prevent CREB phosphorylation, whereas PKA inhibition will not. CREB phosphorylation of CAL-62 cells pretreated with U0126 and then treated with either SB203580 or H-89 was assessed by Western blot. As expected, the inhibition of p38 phosphorylation (Fig. 5c, lower panel) led to a dose-dependent decrease in CREB phosphorylation (Fig. 5c, up panel), whereas PKA inhibition had no effect on CREB phosphorylation (Fig. 5d). This finding prompted us to test whether the specific inhibition of p38 phosphorylation could affect both cellular proliferation and L-GILZ expression. Increasing the dose of SB203580 neither inhibited cellular proliferation nor L-GILZ upregulation (Fig. 5e, f), whereas U0126 inhibited cellular proliferation and upregulated L-GILZ in a dose-dependent manner (Fig. 5e, f).

To further confirm the role of p38 activation in CREB phosphorylation, we treated 8505C cells with PLX4032 and increasing doses of the p38 inhibitor, SB203580. The inhibition of p38 at the highest dose of SB203580 (data not shown) resulted in the inhibition of CREB phosphorylation (Fig. 5g). These data suggest a likely association between hyperactive MAPK cascade inhibition, p38 phosphorylation, CREB activation, and L-GILZ upregulation.

### L-GILZ inhibits tumor cell growth

**Fig. 6:**
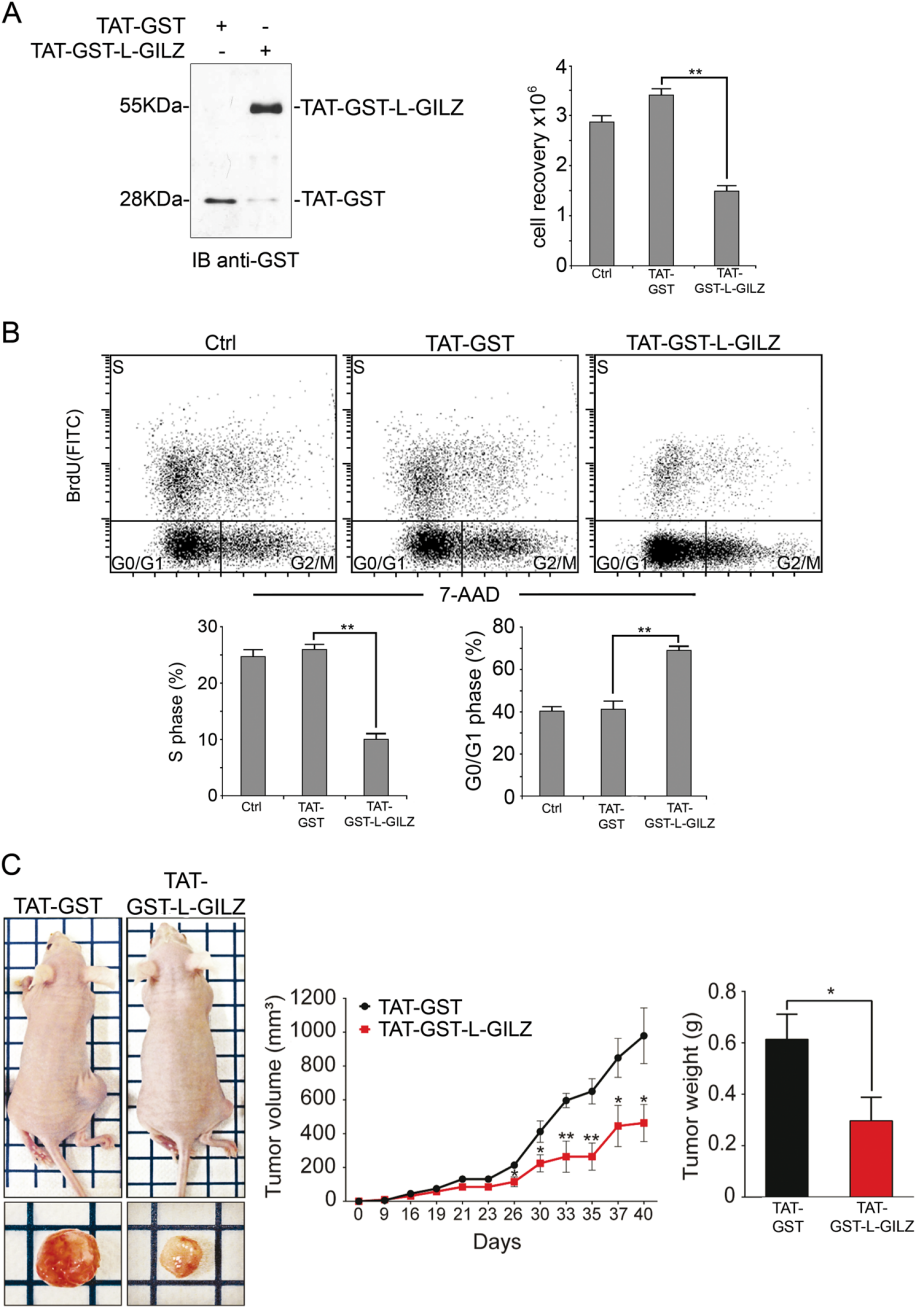
L-GILZ inhibits anaplastic thyroid cancer cell proliferation and tumor growth. The 8505C cells were transfected with either TAT-GST-L-GILZ or control TAT-GST recombinant proteins. A Western blot of the transfected recombinant proteins is shown (**a**, left). Cellular proliferation was assessed by cell counting (**a**, right) and BrdU incorporation (**b**). Representative scatter plots of log FITC anti-BrdU staining versus the total DNA content (red staining) are shown. Bottom histograms show the percentage of cells in the S or G_0_/G_1_ phase. **Significant differences between TAT-GST-L-GILZ-transfected and TAT-GST-transfected cells. **c** The 8505C cells (1 × 10^7^) were subcutaneously injected into the right flank of nude mice (six individuals per group). After 24 h, mice were intraperitoneally inoculated with TAT-GST-L-GILZ or TAT-GST (the control) fusion proteins on alternate days. The tumor volume was determined in vivo at the indicated time points with an external caliper. Mice were sacrificed 40 days after the cells were injected. The tumors were excised, imaged, and weighed. *,**TAT-GST-L-GILZ- versus TAT-GST-treated mice

Finally, we addressed whether L-GILZ is only implicated in mediating antiproliferative activity of MAPK inhibitors or directly inhibits cellular proliferation in vitro and tumor development in vivo. To this end, the proliferation of 8505C cells overexpressing L-GILZ was evaluated. The transactivator of transcription (TAT)-glutathione-*S*-transferase (GST)-L-GILZ fusion protein^6^ was transfected into L-GILZ-deficient 8505C cells and the amount of protein that was transfected was controlled by Western blot (Fig. 6a, left). Cellular proliferation was then evaluated using trypan blue exclusion and 5-bromo-2-deoxyuridine (BrdU) incorporation assays. The exclusion assay indicated that 72 h after transfection, there was a statistically significant reduction in the number of viable cells (*p* < 0.01) in the TAT-GST-L-GILZ-transfected cells compared to the control TAT-GST-transfected cells (Fig. 6a, right). Moreover, similar results were obtained when proliferation was evaluated through the incorporation of BrdU. Indeed, Fig. 6b shows that TAT-GST-L-GILZ-transfected cells displayed a reduction in BrdU incorporation compared to the control cells, which corresponded to a significant decrease (*p* < 0.01) in the percentage of cells in the S phase and an increased number of cells in the G_0_/G_1_ phase 72 h after transfection.

Next, we examined whether L-GILZ affects tumor cell growth. Nude mice were subcutaneously injected with 8505C cells in the flank and then treated intraperitoneally with either TAT-GST-L-GILZ or a TAT-GST-fusion protein on alternate days. The tumor size was monitored for 40 days, when the mice were sacrificed, and the tumors were excised and weighed. Images were taken of representative tumors. We found a significant decrease in the average tumor volume, which was already visible and statistically significant 26 days after commencing treatment with the fusion protein. In addition, the reduced tumor weight (51.78% tumor weight inhibition) in the mice injected with TAT-GST-L-GILZ compared to mice injected with TAT-GST (Fig. 6c) suggests that L-GILZ affects the cellular proliferation and tumor growth of anaplastic thyroid cancer cells.

## DISCUSSION

L-GILZ regulates spermatogonia survival, as well as differentiation, myogenesis, and cancer cell proliferation;^1,4,5,7^ however, the many roles of L-GILZ have not been extensively defined due to its interactions with several signaling proteins, which lead to multiple cellular outcomes. For example, L-GILZ regulates spermatogenesis and myogenesis by inhibiting Ras^4^ and MyoD^1^, respectively. Furthermore, L-GILZ interacts with p53 and MDM2, leading to the activation of p53 and p53-dependent antiproliferative and pro-apoptotic pathways that limit tumor cell growth^5^. The functional interactions of L-GILZ with various modulatory signaling proteins involved in cellular proliferation, differentiation, and apoptosis indicates its probable involvement in human diseases, particularly cancer. Since L-GILZ may be a potential target for inhibiting cancer cell proliferation, we analyzed its expression and function in thyroid cancer cells at diverse stages of differentiation and with different proliferative potential.

Similar to other types of human cancers, the initiation and progression of thyroid cancer results from genetic and epigenetic alterations involving mutations and/or the aberrant activation of signaling pathways critical for controlling cell differentiation and proliferation^9,11^. In thyroid cancer, point mutations in Ras and BRAF genes, as well as rearrangements of the RET gene (RET/PTC), lead to the constitutive activation of MAPK pathways that initiate and/or sustain thyroid tumorigenesis^19^. Moreover, more than one genetic defect may occur, resulting in multiple abnormally activated signaling pathways contributing to the development of aggressive forms of thyroid cancer^17,40^.

In the present study, we have shown that L-GILZ is strongly expressed in highly differentiated and slowly proliferating thyroid cancer cells, but not in less-differentiated, more proliferative, anaplastic thyroid cancer cells; an observation made in both cell lines and patient specimens (Fig. 1). Additionally, based on the two lines of evidence, L-GILZ may play a regulatory role in proliferation: (1) its overexpression in L-GILZ-deficient thyroid cancer cells inhibited their proliferation (Fig. 6a, b), while its silencing in TPC-1-differentiated thyroid cancer cells increased their proliferation (Figs. 3o–r); and (2) its injection into nude mice reduced the growth of xenografts (Fig. 6c). Moreover, L-GILZ was implicated in the antiproliferative effect of MAPK kinase inhibitor drugs (Fig. 3).

BRAF-transformed thyroid cancer cells respond to treatment with PLX4032, a selective inhibitor of BRAF^V600E^ kinase, which inhibits MAPK-dependent cell proliferation^41^. We found that blocking BRAF activity in the 8505C thyroid cell line carrying BRAF^V600E^ resulted in the inhibition of cellular proliferation associated with the upregulation of L-GILZ (Figs. 2 and 3). The ATC cell line, CAL-62, which carries a hyperactivating Ras mutation, did not respond to PLX4032 treatment (Fig. 2). However, when U0126, which targets and inhibits MEK1/2 downstream of BRAF^32^, was supplied to CAL-62 cells, cell proliferation was reduced and L-GILZ expression was increased (Fig. 4). Thus, in both cell lines, reduced proliferation was accompanied by the upregulation of L-GILZ expression, strongly indicating that L-GILZ attenuates thyroid cancer cell proliferation. Indeed, L-GILZ silencing reversed the antiproliferative activity of MAPK inhibitors at distinct pathway points, such as BRAF in BRAF-hyperactive 8505C cells and MEK in Ras-transformed CAL-62 cells (Fig. 3). Therefore, we hypothesize that activating mutations in *Ras* or *BRAF* sustain the constitutive activation of MAPK signaling and may negatively regulate L-GILZ expression. Thus, the inhibition of the MAPK pathway may alleviate this feedback mechanism and promote L-GILZ expression or, alternatively, may promote the activation of transcription factors responsible for the upregulation of L-GILZ. We identified numerous putative binding sites for transcription factors potentially related to the MAPK pathway and L-GILZ transcription.

The cAMP/PKA/CREB pathway plays a pivotal role in both benign and malignant thyroid tumors^18,34,42^, and CREB is involved in the differentiation and proliferation of thyroid cells. Mice expressing dominant-negative CREB experience inhibited follicular thyroid cell differentiation and growth^43^, while a gain-of-function CREB mutant induces in vivo cell differentiation^35^. However, the amplification of CREB activation has been invoked to explain thyroid hyperplasia in mice with a knock-in for *Ccdc6*^*−ex2*^, a gene found to be rearranged with RET in PTC^44^, or sustained cellular proliferation due to the CD44 receptor^45^ or transmembrane serine protease 4^46^, both of which are overexpressed in thyroid cancer cells.

We suggest a novel role for CREB in inducing the transcription of L-GILZ, a protein exhibiting antiproliferative activity on thyroid cancer cells. The PLX4032-mediated or U0126-mediated inhibition of MAPK signaling induced late CREB phosphorylation in both 8505C and CAL-62 cell lines (Fig. 5); this effect corresponded to the transcriptional activity of the *L-GILZ* promoter, which contains putative CREB-binding sites (Fig. 4c–e). Therefore, phosphorylated CREB contributes to L-GILZ expression regulation following MAPK signaling inhibition.

Previous studies have revealed that chronic exposure to PLX4032 of BRAF^V600E^ melanomas induces hyperactivation of the PI3K/Akt/CREB signaling pathway^47^. Activated CREB then upregulates adipocyte enhancer-binding protein 1, leading to nuclear factor-κB activation. This mechanism appears to underlie melanoma resistance to PLX4032^47^. In contrast, we demonstrated that when the 8505C thyroid cancer cell line carrying BRAF^V600E^ is treated with PLX4032, CREB activation is critically related to the drug’s antiproliferative effects via L-GILZ, which also exerts antiproliferative activity. Therefore, targeting BRAF^V600E^ with PLX4032 induces CREB phosphorylation and results in differential biological outcomes, depending on the tumor type and treatment duration. Moreover, we showed that CREB phosphorylation occurs early after treatment and is generally related to MAPK pathway inhibition in both 8505C cells (BRAF^V600E^) treated with PLX4032, and CAL-62 cells (Ras hyperactivation) treated with U0126.

In our model, CREB phosphorylation appears follow a noncanonical pathway, as we observed its activation in two cell lines exhibiting different genetic defects: (1) an H-Ras mutation that hyperactivates Raf/ERK and PI3K/Akt, leading to activation of the mTOR pathway; and (2) BRAF^V600E^, which activates only the ERK pathway. Therefore, the two cell lines share the same constitutive hyperactivation of the MAPK pathway, whose inhibition results in reduced cell proliferation, regardless of the pharmacological inhibitor and cell line type. However, the kinetics of ERK and Akt inhibition differ between the two cell lines, despite the observation that CREB was phosphorylated at the same time points following treatment with the MAPK inhibitor. In addition, ERK phosphorylation, often used as a gauge of MAPK activity, is highly variable depending on experimental conditions. Thus, the expression of genes involved in thyroid differentiation may correlate with a critical threshold of MAPK signaling inhibition^48^. Indeed, we found increased NIS and TSHR protein expression in CAL-62 cells 48 and 72 h following U0126 treatment (data not shown), even though in our in vitro system, ERK phosphorylation resulted not more inhibited.

**Fig. 7:**
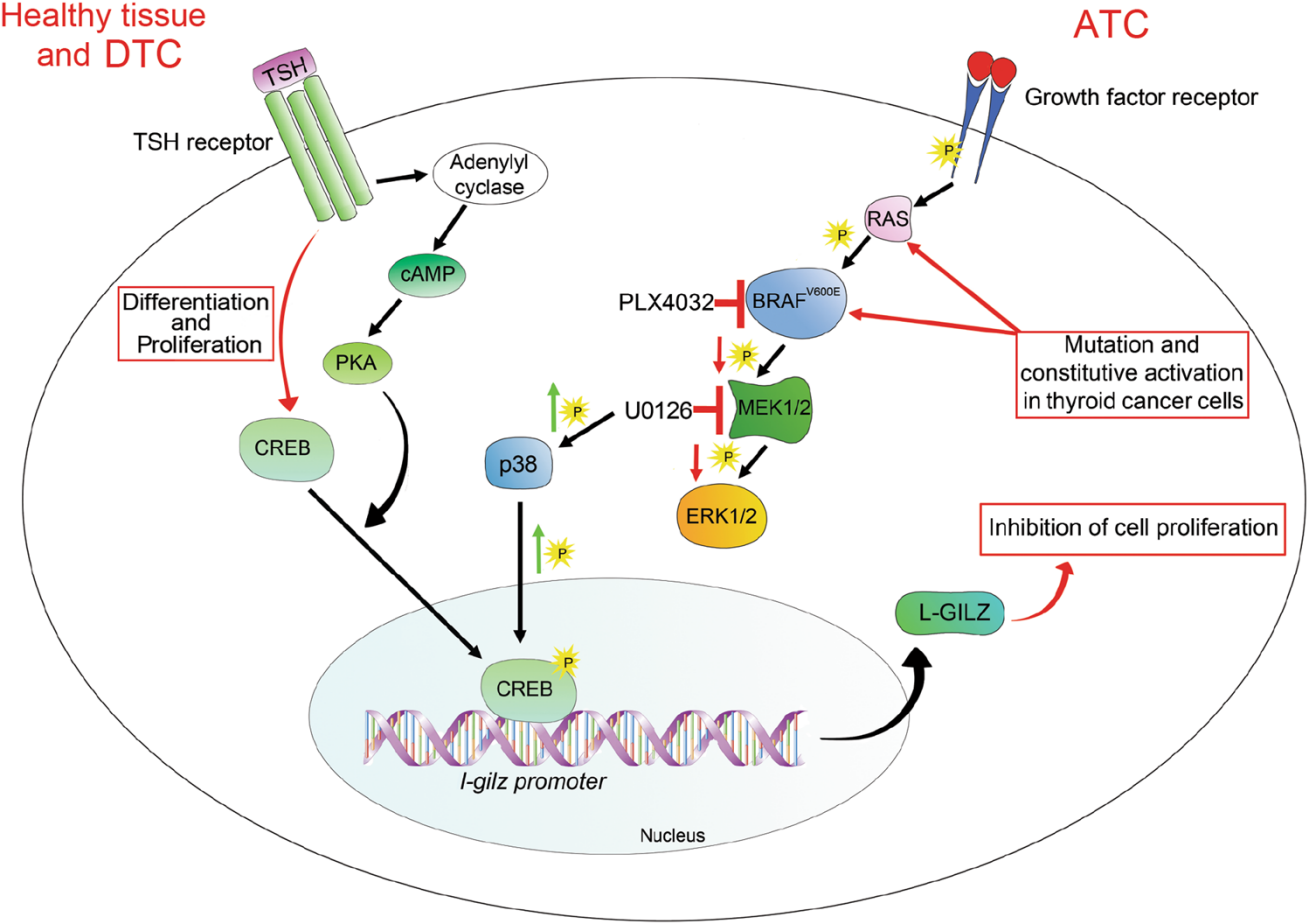
Model of L-GILZ upregulation in anaplastic thyroid cancer cells treated with MAPK inhibitors. In healthy thyroid cells and many differentiated thyroid carcinomas (DTC), TSH triggers TSH receptor/cAMP/PKA pathways thus activating CREB and regulating thyroid cell differentiation and proliferation. In anaplastic thyroid carcinoma (ATC), mutations in *Ras* or *BRAF* induce the constitutive activation of MAPK signaling, leading to transcription factor activation and production of proliferative proteins. Pharmacological inhibition of hyperactivated BRAF or MEK induces the inhibition of MAPK activity, as well as phosphorylation of p38, which leads to CREB activation, increase of L-GILZ transcription through CREB binding to the *L-GILZ* promoter, and the upregulation of L-GILZ protein expression. Ultimately, L-GILZ contributes to the antiproliferative effects of MAPK inhibitors

Our experimental evidence indicates a role for p38 in CREB phosphorylation and L-GILZ upregulation. For example, U0126 limited proliferation and induced p38 and CREB phosphorylation, as well as L-GILZ expression in CAL-62 cells. The inclusion of a p38 inhibitor reversed these effects (Fig. 5). Therefore, we conclude that the pharmacological inhibition of MAPK signaling in undifferentiated thyroid cancer cells carrying a BRAF or Ras mutation induces p38 phosphorylation, which subsequently activates CREB and leads to L-GILZ transcriptional activation. L-GILZ then contributes to the antiproliferative activity of drugs targeting MAPK pathways (Fig. 7).

Finally, if L-GILZ is expressed in more differentiated thyroid tumors, it is reasonable to propose that it directly inhibits thyroid cancer cell proliferation and reduces tumor growth. In fact, TAT-GST-L-GILZ fusion protein inhibited cellular proliferation in an anaplastic thyroid cell line and when inoculated into mice that had been subcutaneously injected with anaplastic thyroid cancer cells, the size and weight of the tumor was reduced (Fig. 6). The discovery that L-GILZ, when intraperitoneally inoculated, retards the growth of 8505C flank xenograft tumors in nude mice is of particular relevance. This is both because it is a further demonstration of the antiproliferative activity of L-GILZ, and suggested therapeutic efficacy, in terms of the bioavailability and delivery of the biologically active TAT-GST-L-GILZ-fusion protein. Thus, it may be that L-GILZ has potential as a therapeutic agent in cancer treatment, regardless of its ability to mediate the effects of MAPK inhibitors. Developing this potential might increase the therapeutic options for patients with tumors resistant to current treatments.

## Materials and methods

### Cell lines, patient samples, treatment compounds, and animals

Human thyroid cell lines from anaplastic carcinomas (CAL-62, C643, and 8505C), papillary carcinomas (BC-PAP), or follicular carcinomas (FTC-133 and TPC-1) were purchased from DSMZ (Braunschweig, Germany) or from ECACC (Salisbury, UK). These cell lines were maintained as previously described^49^. HEK 293 cells were grown in Dulbecco’s modified Eagle's medium containing 10% fetal bovine serum, 100 U/mL penicillin, and 100 μg/mL streptomycin, in a humidified atmosphere at 37 °C and 5% CO_2_. All cell lines were tested for mycoplasma contamination. Surgical specimens from thyroid cancer patients were also obtained. The study was approved by the local medical ethics committee, and written informed consent was obtained from the participants prior to their inclusion in the study. Sorafenib, vemurafenib (PLX4032), U0126, SB203580, and forskolin were purchased from Cell Signaling Technology (Danvers, MA, USA), and H-89 was obtained from Abcam (Cambridge, UK).

Seven-week-old female athymic mice (Nude-Foxn1nu, ENVIGO, Udine, Italy) were acclimatized in a pathogen-free room for 2 weeks with free access to commercial rodent feed and water. Animal care was in compliance with regulations in Italy (D.M. 116192), Europe (O.J. of E.C. L 358/1 12/18/1986), and the United States (Animal Welfare Assurance No. A5594-01, Department of Health and Human Services). For mouse xenograft studies, 1 × 10^7^ 8505C cells were inoculated subcutaneously into the right flank. Tumor growth was monitored using electronic digital calipers. Tumor volume (mm^3^) was calculated as (length x width^2^)/2. Mice were sacrificed 40 days after cells injection and tumors were excised and weighed.

### *L-GILZ* promoter analysis and plasmid construction

The promoter region of hL-GILZ (−1542 bp starting from exon 1) was analyzed using Matinspector software algorithms (Genomatix, München, Germany) for the putative binding sites of transcription factors (e.g., CREB).

The promoter region of hL-GILZ (1562 bp, FULL promoter) and its deleted mutant fragment (648 bp, ΔCREB) were amplified by PCR using primers: F, 5′-AGTTGACGGTACCTGAATGAAGGAGCCGATGCG-3′ and R, 5′-GTCCTGAAGCTTAAGTGTGAGCGGGGATTGG-3′, and F, 5′-AGTTGACGGTACCGCTACAACACTTGGGGCTCT-3′ and R, 5′-GTCCTGAAGCTTAAGTGTGAGCGGGGATTGG-3′, respectively. Amplicons were inserted into the pGL4.71 *Renilla reniformis* vector (Promega, Madison, WI, USA).

The TAT-GST-L-GILZ vector was obtained by cloning the hL-GILZ open reading frame into TAT-C to produce a TAT-GST-L-GILZ fusion protein as previously described^6^.

### Transfection and luciferase assay

The 8505C cells were transfected with either the FULL promoter or the ΔCREB construct using ViaFect Transfection reagent (Promega) and treated with DMSO (dimethyl sulfoxide) or PLX4032 (5 and 0.5 μM, respectively) for 48 h. HEK 293 cells were transfected with either the FULL promoter or ΔCREB construct together with the Y/F CREB gain-of-function mutant (gift from Dr. Montminy, Salk Institute for Biological Studies, La Jolla, CA, USA)^35^. A luciferase assay on the transfected cells was performed using the Dual-Luciferase Reporter Assay System (Promega) according to the manufacturer’s instructions. *Photinus pyralis* was used as an internal control.

The TAT-GST-L-GILZ fusion protein was administrated to 8505C cells (20 μg/mL, 3 × 10^5^ cells per 10-cm dish), and proliferation was measured after 72 h using a trypan blue exclusion test and a BrdU cell proliferation assay kit (BD Pharmingen, San Diego, CA, USA) following the manufacturer’s instructions. Beginning one day after the start of the experiment, TAT-GST-L-GILZ or TAT-GST (the control; 0.4 mg/kg) were administered intraperitoneally to nude mice every other day for 40 days.

### Western blotting

Proteins from the cell lysates of treated or untreated cells were separated using sodium dodecyl sulfate-polyacrylamide gel electrophoresis and assessed by Western blotting. The primary antibodies were specific to L-GILZ (eBioscience, Thermo Fisher Scientific, Waltham, MA, USA), CREB, p-CREB, ERK1/2, p-ERK1/2, Akt, p-Akt, p38, p-p38, GST (Cell Signaling), and β-tubulin (Sigma-Aldrich, Saint Louis, MO, USA). Secondary antibodies were labeled with horseradish peroxide (Pierce, Thermo Fisher Scientific). Complexes were revealed by enhanced chemiluminescence in accordance with the manufacturer’s instructions (Millipore, Billerica, MA, USA). Band signal intensities of the Western blot films were determined using the ImageJ software. Expression levels were normalized to that of β-tubulin, and phosphorylated protein expression levels were normalized to the total protein expression levels.

### RNA interference

The post-transcriptional silencing of the L-GILZ gene was induced by double-stranded RNA interference using the Trilencer-27 siRNA knockdown duplexes kit (Origene, Rockville, MD, USA) according to the manufacturer’s instructions. Briefly, CAL-62 and 8505C cells were transfected with either L-GILZ or scrambled control siRNA in the presence of siTran transfection reagent in culture medium 5 h before U0126 or PLX4032 treatment for 48 h. The cells were then controlled for L-GILZ expression by real-time quantitative reverse transcription-PCR (qRT-PCR), stained with propidium iodide (PI) for flow cytometry analysis, or plated for a clonogenic assay.

### Clonogenic and PI assays

CAL-62 and 8505C cell lines were transfected with either L-GILZ or scrambled control siRNA and treated with 5 μM PLX4032 (8505C) or 10 μM U0126 (CAL-62). After 48 h, 1000 cells were plated in triplicate in 10-cm dishes, incubated at 37 °C for 10 to 12 days, and stained with 0.5% (w/v) crystal violet. The cell culture dishes were photographed for visual analysis, and colonies with diameters of 0.002–0.04 in^2^ were counted using ImageJ. The cell cycle profiles were analyzed by flow cytometry to determine the nuclear DNA content via PI staining as previously described^50^.

### qRT-PCR

Total RNA from cell lines and patient samples was isolated using Trizol reagent (Invitrogen, Thermo Fisher Scientific), and the generation of cDNA was performed in triplicate using a QuantiTect Reverse Transcription kit (Qiagen, Hilden, Germany). All reactions were performed using an ABI-7300 Real-Time Cycler, and amplification was achieved using a TaqMan Assay (Hs00933671 for hL-GILZ and 4326321E for HPRT1, the control, Thermo Fisher Scientific). The ΔΔCt method was used to determine the expression level of hL-GILZ.

### ChIP assay

Assays were performed as described previously^51^. Briefly, CAL-62 cells, untreated or treated with U0126 (10 μM for 48 h), were fixed in 1% paraformaldehyde and sonicated on ice. The lysates were precleared and incubated overnight at 4 °C with anti-p-CREB or control rabbit IgG antibodies (Cell Signaling Technology). Immunocomplexes were collected using a ChIP Assay kit (Millipore), and qPCR analysis was performed using Power SYBR Green PCR Master Mix (Applied Biosystems, Thermo Fisher Scientific). The results were analyzed using the percent input method. The following primers were used for the ChIP analysis of the p-CREB-binding site (CREB1) of the hL-GILZ promoter: F, 5′-CAGAGGAGGGCTTTCTTTCTTCTT-3′ and R, 5′-CCCGGCCTCTTACTTCATTCT-3′.

### PKA and p38 activity

Activation of PKA was evaluated using a PKA activity kit (Abcam) following the manufacturer’s instructions. Briefly, CAL-62 cells were treated with U0126 (10 μM), and 100 ng protein lysates were used for an ELISA (enzyme-linked immunosorbent assay) using a peptide as the PKA substrate and an antibody recognizing each phosphorylated substrate. CAL-62 cells pretreated with U0126 for 48 h were treated with either SB203580, a p38 inhibitor, or H-89, a PKA inhibitor for 24 h. Subsequent CREB phosphorylation was assessed by Western blot. The cells were also treated with forskolin (15 μM) alone or forskolin and H-89. Cells treated with active PKA (100 ng) were used as the positive control. The 8505C cells pretreated with PLX4032 for 48 h were treated with SB203580 for 24 h prior to performing a Western blot analysis with anti-p-CREB and CREB antibodies.

### Statistics

Each experiment was performed a minimum of three times. Due to the non-normal distribution of the data, non-parametric tests (e.g., Kruskal–Wallis analysis of variance) were used for statistical evaluation. Individual group means were compared using a Student’s unpaired *t* test. Statistical tests for thyroid cancers from patients were performed according to a Mann–Whitney *U* test. Differences were considered statistically significant according to the following criteria: *p  <  0.05; **p <  0.01; ***p <  0.001.
